# Observations in Anesthesia

**Published:** 1899-11

**Authors:** 


### Observations in Anesthesia.

This paper, written by a trained nurse, gives the results of some 3,500 anesthesias in St. Mary's Hospital, Rochester, Minn., and she believes that the following conclusions are warranted: That the simple Esmarch mask is superior to any other inhaler now in use; that ether should be given with more air than was formerly supposed necessary; that patients revive quickly with plenty of air and withdrawal of the anesthetic. The main thing in giving an anesthetic is not to feel hurried and not to crowd either ether or chloroform. Avoid the frequent use of gags and tongue forceps. Do not rely on any one danger signal, but carefully watch all symptoms.—*Northwestern Lancet.*

